# Deep ocean mineral water accelerates recovery from physical fatigue

**DOI:** 10.1186/1550-2783-10-7

**Published:** 2013-02-12

**Authors:** Chien-Wen Hou, Yung-Shen Tsai, Wei-Horng Jean, Chung-Yu Chen, John L Ivy, Chih-Yang Huang, Chia-Hua Kuo

**Affiliations:** 1 Laboratory of Exercise Biochemistry, Taipei Physical Education College, Taipei, Taiwan; 2 Department of Anesthesiology, Far Eastern Memorial Hospital, New Taipei, Taiwan; 3 Department of Kinesiology and Health Education, University of Texas at Austin, Austin, Texas, USA; 4 Graduate Institute of Basic Medical Science, China Medical University, Taichung, Taiwan; 5 School of Chinese Medicine, College of Chinese Medicine, China Medical University, Taichung, Taiwan; 6 Department of Biotechnology, Asia University, Taichung, Taiwan

**Keywords:** Deep seawater, Origin of life, Trace elements, Hydrothermal vent hypothesis

## Abstract

**Background:**

Deep oceans have been suggested as a possible site where the origin of life occurred. Along with this theoretical lineage, experiments using components from deep ocean water to recreate life is underway. Here, we propose that if terrestrial organisms indeed evolved from deep oceans, supply of deep ocean mineral water (DOM) to humans, as a land creature, may replenish loss of molecular complexity associated with evolutionary sea-to-land migration.

**Methods:**

We conducted a randomized, double-blind, placebo-controlled crossover human study to evaluate the effect of DOM, taken from a depth of 662 meters off the coast of Hualien, Taiwan, on time of recovery from a fatiguing exercise conducted at 30°C.

**Results:**

The fatiguing exercise protocol caused a protracted reduction in aerobic power (reduced VO_2max_) for 48 h. However, DOM supplementation resulted in complete recovery of aerobic power within 4 h (P < 0.05). Muscle power was also elevated above placebo levels within 24 h of recovery (P < 0.05). Increased circulating creatine kinase (CK) and myoglobin, indicatives of exercise-induced muscle damage, were completely eliminated by DOM (P < 0.05) in parallel with attenuated oxidative damage (P < 0.05).

**Conclusion:**

Our results provide compelling evidence that DOM contains soluble elements, which can increase human recovery following an exhaustive physical challenge.

## Introduction

A living organism can be regarded as a gathering of diverse molecules originating from the earth that works cooperatively to decrease entropy against the catabolic stresses from an ever-changing environment. Deep ocean mineral water (DOM) has been suggested to contain the primordial source of chemical components contributing to the creation of life [1,2]. Besides the major minerals, more than 70 trace elements existing in the ocean water have been documented [3]. The question regarding how many chemical components are necessary or required to support the best complexity of human life is not completely defined.

Presently, there is no information as to the effect of DOM on the physiological function of animals or humans following extreme environmental or physiological challenges. The most consistent observations reside around the anti-atherogenic effects of DOM against dietary challenges [4-7]. Compared to desalinated surface ocean water with a similar profile of major minerals (magnesium, potassium, calcium, sodium, chloride, and sulfate ions), desalinated DOM has been found to have a much superior effect on preventing the development of atherosclerosis in rabbits challenged with a high cholesterol diet [4]. This result suggests that the highly diverse trace elements found in DOM are responsible for its anti-atherogenic capabilities and have significant physiological effects on terrestrial animals. It is possible the surface waters of the oceans where sunlight is permeable are devoid of these important trace elements as a result of the photosynthetic activity of many marine organisms [8].

Due to environmental limitations marine and terrestrial organisms rely on different nutritive sources to maintain life [9]. Paleobiological evidence, however, strongly suggests terrestrial life evolved from marine ancestor [10]. Although sharing common cellular constituents with marine organisms, terrestrial survivors had to acquire alternative nutritive sources from the land to compensate for the loss associated with ancient sea-to-land migration. We proposed that if deep oceans contain the evolutionary preferred constituents for terrestrial descendents, DOM supplementation can be complementary to achieve the best biological complexity for land animals. To test this hypothesis, we conducted a human study in which we determined the time required for physical performance to recover after a dehydrating exercise when desalinated DOM or placebo drink was supplied for rehydration.

## Methods

### Subjects

Subjects taking alcohol, medication, or nutritional supplements were excluded from the study. Twelve healthy male volunteers (age 24 ± 0.8 y; height 171.8 ±1.5 cm; weight 68.2 ±2.3 kg; VO_2max_ 49.7 ± 2.2 ml · kg^−1^ · min^−1^) were enrolled as participants in the study. Baseline VO_2max_ were measured 72 h before the beginning of the study. Written informed consent was obtained after explanation of the purpose and experimental procedures of the study. This study was approved by the appropriate university Institutional Review Boards and performed in accordance with principles of the Declaration of Helsinki.

### Drink

The desalinated DOM, taken from the West Pacific Ocean (662 meters in depth), was kindly provided by Taiwan Yes Deep Ocean Water Co., Ltd. (Hualien, Taiwan). DOM was filtered by a micro-filter (removal of microorganism) and an ultra-filter (removal of macromolecule and virus) before use. Molecules sized above 1.5 KD were removed after the two filtration procedures. To mask the taste difference between DOM and placebo, the same amount of sucrose, artificial flavors, citrate, citrus juice, calcium lactate, potassium chloride, vitamin C, and mixed amino acids was added to each. Tap water purified by reverse osmosis process was used for making the placebo drink.

### Experimental design

An exercise-challenge protocol used by Nose et al. was modified for this study [11]. Subjects were required to run on a motorized treadmill at 40% VO_2max_ at a room temperature of 30°C until a body mass decline of 3% (maximal running time: 240 min). During recovery, subjects consumed pure water or DOM containing the ingredients listed above at an amount equivalent to 1.5 fold of their body mass loss [12]. Water supplements were evenly divided into 4 sub-supplements and ingested at 30-minute intervals. Measures of physical performance (aerobic power and lower-body muscle power), physiological stress, and muscle damage were determined 4, 24, and 48 h during the recovery period. To control for possible confounding effects of individual variation, a randomized double-blind crossover design was employed with trials spaced 7 d apart.

### Physical performance

Aerobic power (maximal oxygen consumption, VO_2max_) and peak lower-body muscle power were the physical performance measures selected for determining the degree of physical fatigue recovery. VO_2max_ was evaluated by the Bruce graded treadmill running protocol. This protocol consists of a 5-min warm up and incremental increases in speed and grade every 3 min until exhaustion. Verification that VO_2max_ was achieved was a Respiratory Exchange Ratio (RER) greater than 1.1 and a plateau in VO_2_ with increasing workload. Samples of expired gases were analyzed using a MetaMax3B (Cortex Biophysik, Nonnenstrasse, Leipzing, Germany). Peak lower-body muscle power was assessed using a Bertec force plate (4060-NC2000, Bertec Corporation, Columbus, Ohio, USA) with a sampling rate of 1,000 Hz. Each subject performed 3 repetitions of maximal squat jumps from a 90° knee flexion angle to full extension. Subjects were signaled when to jump by a light placed 2 meters in front of them at eye level. There was a one-minute rest between jumps. Velocity and power of each jump was calculated from vertical ground reaction forces (V_GRF_) according to the impulse-momentum theorem (V_GRF_ × time = body mass times ΔV, ΔV is the change in vertical velocity) (Innovative Sports Training, Inc, Chicago, IL, USA). Instantaneous velocity was determined by adding ΔV to the previous time interval, starting at zero at the beginning of the jump. Instantaneous power was derived from the product of V_GRF_ measured by the force plate and the calculated instantaneous velocity [13]. The peak value of instantaneous power during the entire period of each jump was selected as peak power. The peak power values of the 3 jumps were averaged for statistical calculation.

### Biochemical analysis

Venous blood samples were assayed for plasma myoglobin (Immunology Consultants Laboratory, Inc. OR, USA), thiobarbituric acid reactive substances (TBARS) (Cayman Chemical Company, Ann Arbor, MI, USA), cortisol (IBL-America, Inc. MN, USA), erythropoietin (eBioscience, Vienna, Austria), IL-6 (eBioscience, Vienna, Austria), and testosterone (Nova Tec Immundiagnostica GmbH, Dietzenbach, Germany) with enzyme-linked immunosorbent (ELISA) readers (Tecan Genios, Salzburg, Austria). Plama CK was analyzed enzymatically using a bench top DT-60II analyzer (Johnson and Johnson, NY, USA).

### Statistical analyses

All values are expressed as percent of baseline (mean ± standard error). A two-way analysis of variance with repeated measures was used for comparisons between DOM and pure water at specified time points during recovery. A paired t test with Bonferroni’s correction was used to compare treatment differences at each time point. Probability of a type I error less than 5% was considered statistically significant.

## Results

**Figure 1:**
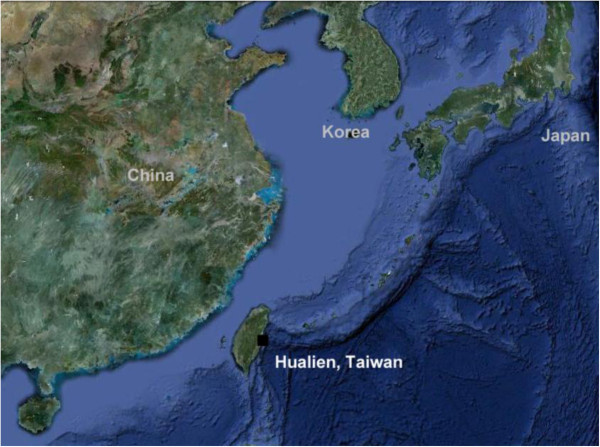
**Geographic location of DOM collection.** The black square designates the site of seawater collection, providing the shortest piping distance from land down to the deep site of the ocean (a depth of 662 meters off the coast of Hualien, Taiwan) along the circum-Pacific belt (known as Pacific Ring of Fire) in East Asia.

**Figure 2:**
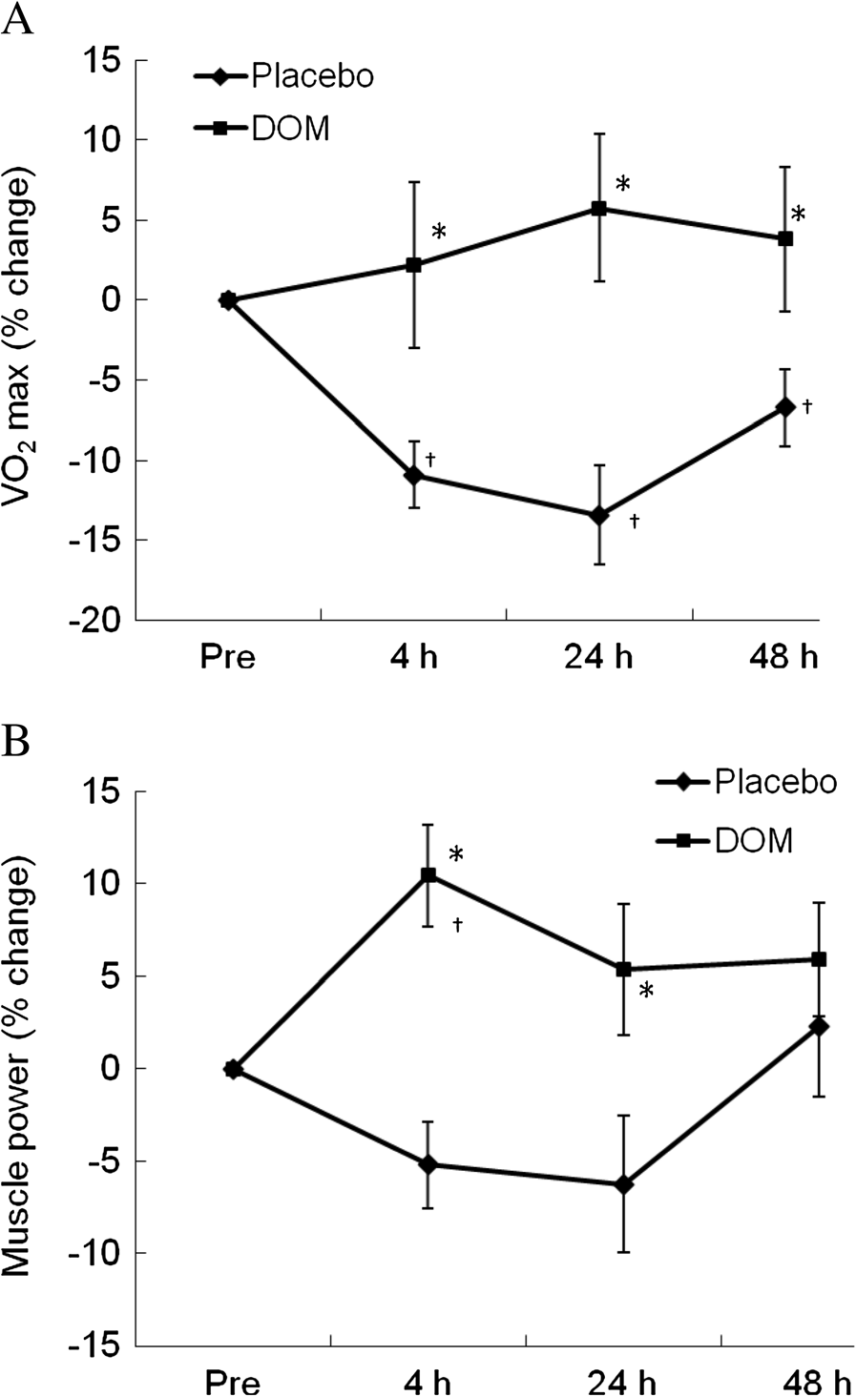
**Human physical performance.** DOM accelerated the recovery of aerobic capacity after a fatiguing exercise (**A**), and increased lower-body muscle power performance (**B**) during recovery. *significance against Placebo, P < 0.05; †significance against Pre, P < 0.05. N. D.: non-detectable.

The geographic location of DOM is illustrated in Figure 1. Concentrations of the minerals and trace elements of DOM are shown in Table 1. Our physical challenge protocol successfully induced a prolonged physical fatigue in aerobic power of our control trial (RO purified water) for 48 h of recovery (Figure 2A, P < 0.05). DOM supplementation completely restored the loss of aerobic power to baseline within 4 h. Lower-body muscle power was not affected by our physical challenge protocol, yet DOM supplementation increased the power performance by ~10% above baseline (Figure 2B) at 4 h and 24 h during the recovery (P < 0.05).

**Table 1:** Minerals and trace elements in deep ocean mineral water (DOM) drink

**Mineral**	**Placebo (mg/L)**	**DOM (mg/L)**
Na	38.3	119
K	75.6	115.6
Ca	53.1	54.6
Mg	3.24	140
Trace element	Placebo (μg/L)	DOM (μg/L)
Li	N. D.	17
Rb	N. D.	16
B	N. D.	1590
Osmolarity	226 (mOsm/L)	249 (mOsm/L)

**Figure 3:**
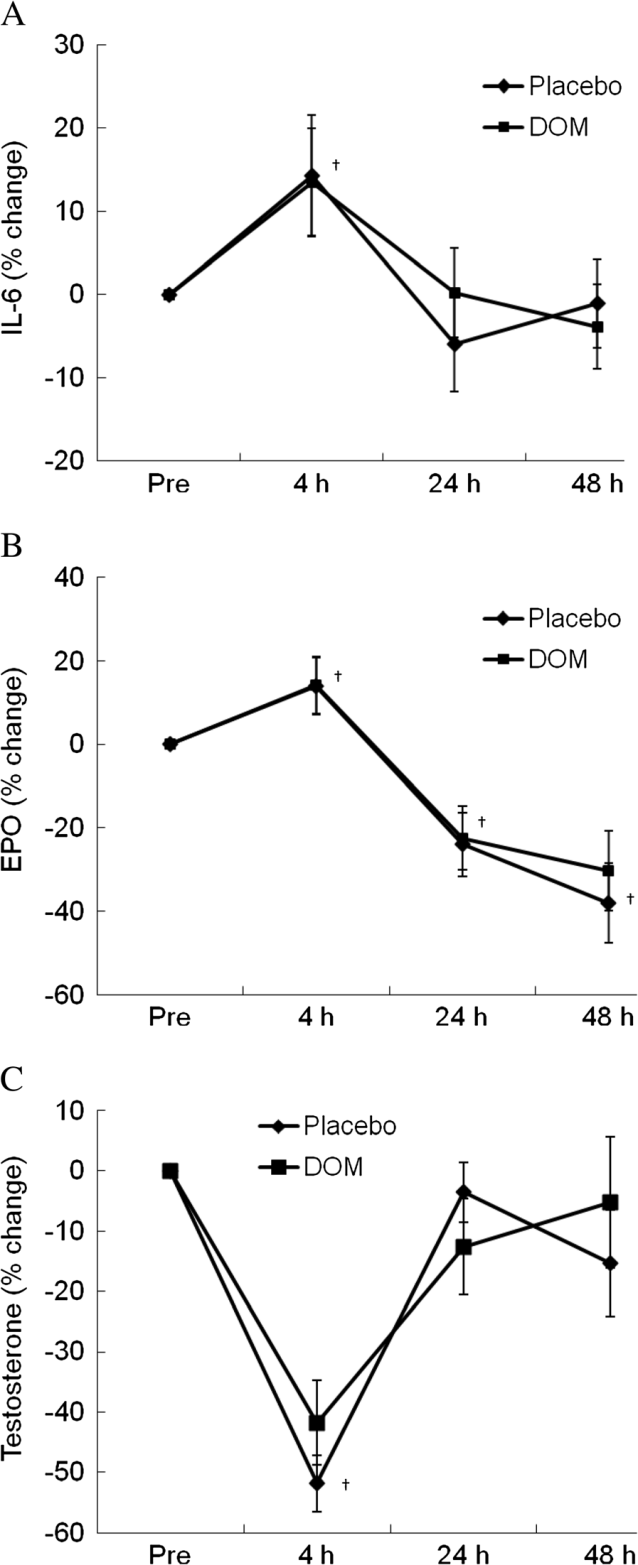
**Stress hormones.** Exercise challenge elevated plasma IL-6 (**A**) and EPO levels (**B**, P < 0.05) for both trials to a similar extent. Testosterone dropped on both trials during recovery (**C**, P < 0.05), and returned to baseline by 24 h during recovery. No group differences in stress hormone responses were found after the physical challenge. †significance against Pre, P < 0.05.

Stress hormone responses are shown in Figure 3 and confirms the same physiological stress produced during each trial. For both control and DOM trials, the exercise challenge temporally elevated plasma IL-6 levels (14%, P < 0.05) at 4 h of recovery to a comparable extent (Figure 3B). This increase subsided to baseline within 24 h. Similarly, we observed a rise in erythropoietin (EPO) of 14% (P < 0.05) at 4 h of recovery for both treatments. By 24 h of recovery, however, EPO had fallen below baseline and was still below baseline at 48 h of recovery (P < 0.05). Both cortisol and testosterone dropped at 4 h during recovery (by 46% and 52%, P < 0.05), and had returned close to baseline by 24 h and 48 h following exercise. Again, there was no treatment differences associated with these hormones.

**Figure 4:**
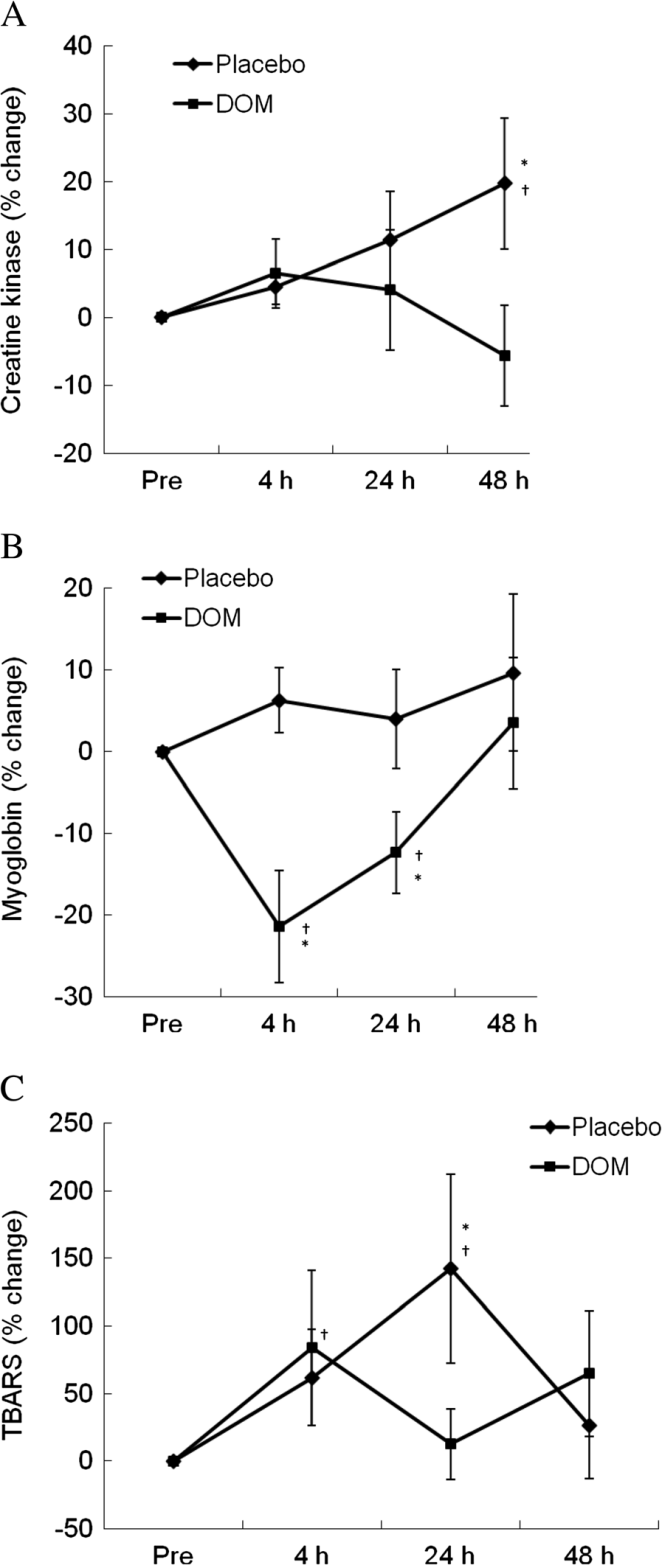
**Muscle damage markers.** Exercise-induced muscle damage was suppressed by DOM, as indicated by attenuated CK (**A**) and myoglobin (**B**) responses during recovery. DOM also attenuated oxidative damage (TBARS) increased by exercise (**C**). *significance against Placebo, P < 0.05; †significance against Pre, P < 0.05.

Plasma CK and myoglobin, known as exercise-induced muscle damage markers [14], are shown in Figure 3. A gradual rise in CK was observed 48 h following exercise in the control trial (Figure 4A), while DOM eliminated this increase (P < 0.05). A marginal increase in myoglobin was observed at 4 h and 24 h following exercise in the control trial, while following the DOM treatment myoglobin was significantly below the control level at 4 and 24 h of recovery (Figure 4B). Results for the oxidative marker thiobarbituric acid reactive substances (TBARS) are shown in Figure 4C. TBARS increased significantly during the control trial at 4 h and 24 h of recovery (P < 0.05), while increasing only at 4 h of recovery during the DOM trial.

## Discussion

In this study, we propose that if terrestrial organisms evolved from deep ocean [10], supply of deep ocean mineral water (DOM) to humans may replenish loss of molecular complexity associated with evolutionary sea-to-land migration, and optimizes the biological fitness. Here, we provide evidence that desalinated DOM, taken from 662 meters below sea-level, can substantially accelerate recovery from physical fatigue in aerobic power and enhance lower-body muscle power after a prolonged bout of dehydrating exercise. This improvement appears to be associated with a complete elimination of exercise-induced muscle damage, suggesting that DOM contains components, which can complement and enhance the molecular and cellular complexity of humans to minimize entropic stress produced during prolonged physical activity in the heat.

The key components of DOM contributing to the observed ergogenic benefits are not exactly known. In the study, the DOM taken from the west rim of the Pacific Ocean is characterized by enriched contents of boron, magnesium, lithium, and rubidium. In DOM the content of boron (1.59 mg/L), which is now considered an essential nutrient for humans, is 5–10 fold that found in human serum (~0.2-0.3 mg/L) [15]. Boron is known to attenuate exercise-induced rise in plasma lactate in animals [16] and to prevent magnesium loss in humans [17]. Serum magnesium concentration and dietary magnesium intake are known correlates of muscle strength [18,19]. Therefore, the minerals and trace elements in DOM may work cooperatively to sustain normal human performance.

The observed effect of DOM on accelerating fatigue recovery is closely associated with the eradication of exercise-induced muscle damage [20,21]. Elevation of these muscle damage markers normally occurs in parallel with increased oxidative damage [22]. Our results on thiobarbituric acid reactive substances (TBARS) fits well with those on markers of muscle damage (P < 0.05). Higher content of magnesium, lithium, and rubidium in DOM may be associated with strengthened antioxidant capability against oxidative stress during post-exercise recovery [23-25]. In animals, lack of magnesium in their diet leads to increased free radical production [26], while magnesium supplementation eliminates free radical production induced by ischemia reperfusion [23] and alcohol drinking [27]. Lithium can increase the free radical scavenging capability in animals [25] and thus help to increase the resilience of a cell against destructive free radical attack [28].

One significant feature of DOM is the enriched rubidium content compared to fresh water. Rubidium concentration increases considerably in seawater as the depth of the ocean approaches 450 meters. The concentration of this trace element in human plasma ranges from 40–310 μg/L [29], about 2.5-20 fold higher than that found in DOM. However, rubidium has a high retention rate in the human body, taking 39-134 days for 50% of infused rubidium to be excreted into urine and feces [30]. Compared to rats fed rubidium, rats fed a rubidium-free diet exhibit higher urea nitrogen in plasma [31], suggesting that rubidium is essential to preserve biological integrity against daily entropic stress. The rubidium concentration in the human brain decreases with age [32], and supplementation of rubidium chloride has been found to increase spontaneous physical activity in animals [33]. Additions of lithium and rubidium into seawater have been shown to increase frequency of movement in jellyfish [34]. The recommended dietary allowance for rubidium has not yet been defined for humans. Rubidium demonstrates interchangeability with potassium in a variety of biological systems meaning that rubidium deficiency can be compensated by supplementation of potassium in many species [35]. Compared to potassium, rubidium may be an evolutionary preferred nutritive source for animals.

The oceans are the largest water reservoirs on earth, which consists of a great diversity of water-soluble chemical components, feeding a vast quantity of marine organisms [8,36]. However, nutrients in the clear ocean surface water have most likely been exhausted by a high rate of photosynthesis [8,37]. Compared to the surface layer of the oceans, DOM may exert greater metabolic benefit, evidenced by its superior action on eliminating oxidative stress and preventing vascular damage in terrestrial animals challenged with a high cholesterol diet [4]. This observation implies that the water-soluble components unique to (or enriched in) DOM may play an important role in supporting metabolic functions of terrestrial animals when they are faced with a various physiological and metabolic challenges.

The limitation of the study is the loci-specific distribution of minerals and trace elements in the ocean, thus preventing us from being able to generalize that DOM from all sites of the world can confer the same ergogenic benefits as presented. Geographic specificity is suggested by a report documenting relatively lower silver, cobalt and nickel concentrations in the North Atlantic Ocean than the other major oceans [38]. Furthermore, the profile of minerals and trace elements is also varied with the depth of the ocean [37,39], and hydrothermal activity and diffusion from bottom sediments can also influence the composition of minerals and trace elements in the ocean waters [40]. Experiments using Antarctic Ocean waters have also suggested that not all deep ocean water will provide comparable biogenic benefits [41].

On the application side, we co nfirm the benefit of acute DOM supplementation on decreasing physical fatigue with elimination of post-exercise oxidative damage. However, it has been reported a diminished training effect when antioxidant was supplemented to trained men [42], suggesting that free radicals may play a role for training adaptation. Thus, whether or not decreasing oxidative stress by DOM supplementation may confer negative effects on exercise training adaptation demands more investigation.

## Conclusion

Our findings demonstrate that desalinated DOM can increase human robustness against an entropic physical challenge, and this positive outcome appears to be associated with its protection against exercise-induced muscle damage. DOM consists of many minerals and trace elements that could not be *de novo* synthesized by the human body. Thus the momentary imbalance between loss and gain of essential minerals and trace elements after prolonged exercise may underlie the delayed recovery from physical fatigue in humans. In line with the “deep ocean life of origin hypothesis”, the results of this study imply that DOM can provide required nutrients for humans that will speed recovery from entropic physical stress.

## Competing interests

The authors declare that they have no competing interests.

## Authors’ contributions

CWH, WHC, YST, CYC, CYH and CHK designed the experiments. CWH and YST performed the experiments. CWH performed the statistical analyses. CWH, JLI, and CHK wrote the manuscript. All authors read and approved the final manuscript.
